# Leading by Example: Arizona’s Bold Step for Safer Catheterization Laboratories to Protect Our Doctors and Nurses

**DOI:** 10.1016/j.jscai.2026.105405

**Published:** 2026-07-14

**Authors:** Carine Werner

**Affiliations:** Arizona State Senator - District 4, Chairwoman of the Arizona Senate Health and Human Services Committee, Scottsdale, Arizona

**Keywords:** catheterization laboratory, policy, radiation, safety

It takes courage to admit when the status quo isn’t good enough, especially in health care, where so much has been accomplished but so much more remains to be done. This legislative session, the Arizona state legislature has advanced bills to encourage broad adoption of enhanced radiation protection devices in the cardiac catheterization laboratories in Arizona hospitals. I am proud to sponsor this legislation because Arizona must choose to lead, not to wait.

This editorial accompanies a call by Society for Cardiovascular Angiography & Interventions (SCAI) for nationwide action on radiation safety.^1^ I am introducing this legislation because we owe the most basic safety protections to those who put themselves on the front lines of medicine every day. We talk a lot about training and attracting new doctors, and it is critical we do so, but we can also do more to protect the health care workers we already have to relieve the existing shortage we face.

## The invisible danger in our most advanced laboratories

Anyone who has walked into a cardiac catheterization laboratory knows the thrill of modern medicine, a place where lives are saved with a tiny wire and a steady hand. But step past the promise, and you see a reality that’s harder to stomach: our most sophisticated spaces are still exposing doctors, nurses, and technologists to levels of radiation that would be considered unacceptable in nearly any other industry.

It’s a problem that wears many faces. Chronic exposure means an elevated risk of cancer and cataracts. The heavy “lead” aprons that are meant to protect can themselves leave a legacy of spinal injuries and careers ended too early due to debilitating injuries.^2^^,^^3^ For female health care workers, the reality of radiation exposure doesn’t just follow them home; it informs life choices, pregnancies, and futures.^4^^,^^5^ The irony couldn’t be starker: the very advances that let us repair hearts through a pinhole have left the people who perform these feats carrying invisible, and too often, lifelong permanent scars.

Recent years have pulled back the curtain on these hazards for the entire country. The Emmy Award-winning PBS documentary “Scattered Denial” did what policy reports alone could never accomplish by exposing the realities of radiation danger in our procedure rooms to the general public. Through the stories of real clinicians, it forced a reckoning not only among hospital administrators and lawmakers but also in every living room that watched. Denial is no longer possible once you see the faces and hear the voices of those carrying the burden.

## Why Arizona? Why now?

Federal regulations around occupational radiation safety exist, but they are a patchwork of inconsistent laws and lag behind the accessible safety measures that exist today. Hospitals do what is required but not always what is necessary. Although professional societies have sharpened the evidence and issued the warnings, action from governing bodies has been slow.

Arizona must step up because we can no longer accept outdated definitions of “reasonable risk” for our health care workers. We have the technology; enhanced radiation protection devices now offer up to 99% reductions in operator exposure compared with traditional aprons or shields, with the potential to eliminate much of the orthopedic burden borne by staff.^6^^,^^7^ Best of all, their efficacy isn’t theoretical; they’ve been proven, rigorously tested, and validated by real-time dosimetry and clinical studies.

We also know that meaningful safety goes beyond gadgets and mandates. Just as important are robust training, enforcement, and a culture of continuous monitoring so that every staff member, from the head of cardiology to the newest nurse, understands how to protect both themselves and their teammates at every moment.

## A moral and legal imperative

To be blunt, reliance on outdated personal protective equipment is no longer acceptable. The ALARA standard (“as low as reasonably achievable”) is the law, not a suggestion. Now that we can do better, we must, or we are in violation of both law and ethics. Failure to implement advances that have been shown to be feasible and effective puts institutions at risk of avoidable harm and liability. It should also prick the conscience of anyone in a position to effect change.

Hospital economics can no longer justify delay; the cost of inaction is measured in cancer diagnoses, ruined backs, and lives changed forever. As regulators, as doctors, as human beings—we are responsible.

## Setting a national standard—not just for today, but for tomorrow

States have always been the laboratories of democracy—testing new solutions, proving what’s possible, and providing models for national progress. With this legislation, Arizona will show that the courage to protect health workers, even ahead of federal mandates or slow-moving national standards, is the truest test of our values. If we succeed, it will not just be Arizona’s catheterization laboratories that become safer. It will be the start of a broader movement—a model that other states, and ultimately the whole nation, can adopt. Standardizing requirements, certification, and monitoring without getting lost in a tangle of inconsistent state and federal rules will move us closer to an America where no doctor or nurse has to choose between healing others and protecting themselves.

## The road ahead: A call to my colleagues

Change won’t be easy. There will be some pushback, citing costs or “adequate” existing protocols. Others will wait for Washington to act. But we know that leadership is about more than waiting your turn.

To my colleagues in medicine, administration, and statehouses across the country: Arizona is forging this path in honor of every practitioner who has spent a career under fluoroscopy expecting their bravery and skill to be enough. Our best science, our best technology, and our best traditions demand nothing less than our full protection—and for once, the path is clear.

It is time we step up, act with urgency, and make it law that those who save lives shouldn’t be forced to risk their own. We have long felt the pinch of doctor and health care worker shortages throughout Arizona, and this small, simple step can provide a meaningful path to protect our doctors, nurses, and technologists. I hope I can count on your support to make this legislation a reality; connect with me at www.wernerforaz.com.

## Declaration of competing interest

The author declared no potential conflicts of interest with respect to the research, authorship, and/or publication of this article.
